# A Food Pyramid and Nutritional Strategies for Managing Nausea and Vomiting During Pregnancy: A Systematic Review

**DOI:** 10.3390/foods14030373

**Published:** 2025-01-23

**Authors:** Mariangela Rondanelli, Simone Perna, Carlo Cattaneo, Clara Gasparri, Gaetan Claude Barrile, Alessia Moroni, Leonardo Minonne, Alessandro Lazzarotti, Francesca Mansueto, Giuseppe Mazzola

**Affiliations:** 1 Department of Public Health, Experimental and Forensic Medicine, University of Pavia, 27100 Pavia, Italy; mariangela.rondanelli@unipv.it; 2 Department of Food, Environmental and Nutritional Sciences, Division of Human Nutrition, University of Milan, 20133 Milan, Italy; simone.perna@unimi.it; 3 Endocrinology and Nutrition Unit, Azienda di Servizi alla Persona “Istituto Santa Margherita”, University of Pavia, 27100 Pavia, Italy; carlo.cattaneo03@universitadipavia.it (C.C.); clara.gasparri01@universitadipavia.it (C.G.); gaetanclaude.barrile01@universitadipavia.it (G.C.B.); alessia.moroni02@universitadipavia.it (A.M.); leonardo.minonne01@universitadipavia.it (L.M.); alessandro.lazzarotti01@universitadipavia.it (A.L.); francesca.mansueto01@universitadipavia.it (F.M.)

**Keywords:** nausea and vomiting in pregnancy (NVP), hyperemesis gravidarum (HG), nutritional strategies, food pyramid, pregnancy nutrition

## Abstract

Nausea and vomiting during pregnancy (NVP) affect up to 85% of pregnant women and usually begin between the 4th and 7th weeks of gestation, and symptoms often peak around the 9th week and generally resolve by the 20th week in most cases, with severe cases termed hyperemesis gravidarum (HG) impacting physical and psychological health. This review aims to provide a structured dietary approach to managing NVP by developing a food pyramid specifically for this population, based on a systematic evaluation of dietary evidence. The findings highlight the beneficial effects of dietary patterns rich in fruits, vegetables, and protein sources in reducing NVP symptoms. Protein intake is shown to alleviate nausea and vomiting by stabilizing gastric motility and addressing nutritional deficiencies. The review also explores the potential benefits of herbal supplements, like ginger and vitamin B6. By integrating these dietary strategies with pharmacological treatments, a more holistic approach to managing NVP can be achieved, enhancing both maternal well-being and fetal health. The proposed food pyramid emphasizes glycemic stability, hydration, and gradual nutrient intake, offering a structured dietary guide for pregnant women experiencing NVP and HG.

## 1. Introduction

Nausea and vomiting in pregnancy is a common condition characterized by varying degrees of nausea and/or vomiting, typically occurring in early pregnancy, affecting between 50% and 80% of pregnant women globally, and persisting into the third trimester in approximately 23.5% of cases [1]. Recent Italian data report an overall prevalence of NVP of 65.5% (with 41.4% experiencing nausea only, 3.9% vomiting only, and 54.6% both) [1].

NVP often begins around 7.2 weeks of gestation and lasts for an average of 10.2 weeks, impacting daily activities, work, and social relationships for over 50% of affected women [1]. Hyperemesis gravidarum (HG), is the most severe form of nausea and vomiting in pregnancy with no universally agreed upon diagnostic criteria [1]. Biomolecular and para physiological mechanisms are closely associated with NVP and HG. Hormones play a crucial role in the development of nausea and vomiting during pregnancy, with key hormonal players, including human chorionic gonadotropin (hCG), progesterone, ghrelin, and serotonin. Human chorionic gonadotropin (hCG), which peaks between the 9th and 12th weeks of pregnancy, is particularly associated with an increase in nausea, as it stimulates the vomiting center in the brainstem, suggesting a causal link [2]. Progesterone also contributes by relaxing the smooth muscle in the gastrointestinal tract, thus slowing gastric motility and exacerbating nausea symptoms. Ghrelin, an appetite-regulating hormone, decreases during pregnancy, especially in cases of HG, potentially reducing appetite and increasing [3]. Serotonin, primarily produced in the intestinal enterochromaffin cells, interacts with receptors that trigger nausea signals to the brain, intensifying symptoms, particularly when the levels of hCG and progesterone are high [4]. Estrogen and cortisol also influence the pathophysiology of NVP. Elevated estrogen levels during pregnancy have been linked to increased sensitivity to odors, a common trigger for nausea, by enhancing olfactory perception and increasing aversions [2]. Additionally, elevated cortisol levels, often associated with stress, can exacerbate gastric discomfort by stimulating the hypothalamic–pituitary–adrenal axis, further activating the nausea reflex [3]. The interplay between these hormones and neurotransmitters, such as dopamine, complicates the regulatory mechanisms controlling appetite and emesis, creating a multifaceted hormonal environment that increases the susceptibility to NVP and HG symptoms. Together, these biochemical factors contribute to a cycle of nausea, diminished appetite, and nutrient malabsorption, which collectively impact both maternal well-being and fetal health [3,5].

Pharmacological treatment for NVP often involves antiemetic medications, such as doxylamine-pyridoxine and ondansetron, which target neurotransmitter pathways associated with the vomiting reflex. Studies have shown these treatments to be effective in reducing severe symptoms, though some antiemetics carry a potential risk for mild side effects [5]. Integrating pharmacological options with dietary strategies may offer a more holistic approach to managing these symptoms in a safe and balanced manner.

An adequate and balanced diet plays a crucial role in managing NVP and HG, as it can help stabilize blood glucose levels and maintain essential nutrient intake. Studies have shown that diets rich in complex carbohydrates, proteins, and essential vitamins can support symptom management and reduce the likelihood of nutritional deficiencies associated with severe nausea [6,7]. However, to date, there are no official guidelines that provide comprehensive recommendations on balanced nutrition and supplementation specifically for managing NVP and HG in the general pregnant population. No established dietary model, such as a dedicated food pyramid, exists that integrates these elements to address NVP and HG on a population-wide basis.

The primary aim of this study is to develop a tailored food pyramid for managing nausea and vomiting during pregnancy (NVP) and hyperemesis gravidarum (HG). The study aims to provide a structured dietary approach by examining the roles of macronutrients, micronutrients, and specific food groups, as well as the effectiveness of herbal supplements, like ginger and vitamin B6, in alleviating symptoms. The goal is to create a comprehensive dietary guide that can support both maternal well-being and fetal health by integrating nutritional strategies with existing pharmacological treatments.

## 2. Materials and Methods

We conducted a systematic review of the literature to identify relevant studies supporting the development of a food pyramid for NVP and HG management. A structured search was performed in the PubMed database using the Bolerian query reported in Supplementary Materials. Inclusion criteria for this review included the following:

1. Every type of article published from 1990 to the present, ensuring the inclusion of contemporary dietary insights;
2. Studies focused on specific nutritional components (e.g., proteins, vitamin B6), individual foods (e.g., chamomile, ginger), or dietary patterns (e.g., Mediterranean diet) relevant to elaborate nutritional recommendations for the prevention and/or management of NVP and HG.

Studies addressing artificial nutrition (e.g., enteral or parenteral nutrition) were excluded to maintain a focus on oral dietary approaches, more suited to a nutritional pyramid. Including studies on enteral or parenteral nutrition would have resulted in a nutritional pyramid tailored to two distinct populations with differing physiological states and nutritional needs, thereby complicating the development of targeted dietary recommendations.

**Figure 1:**
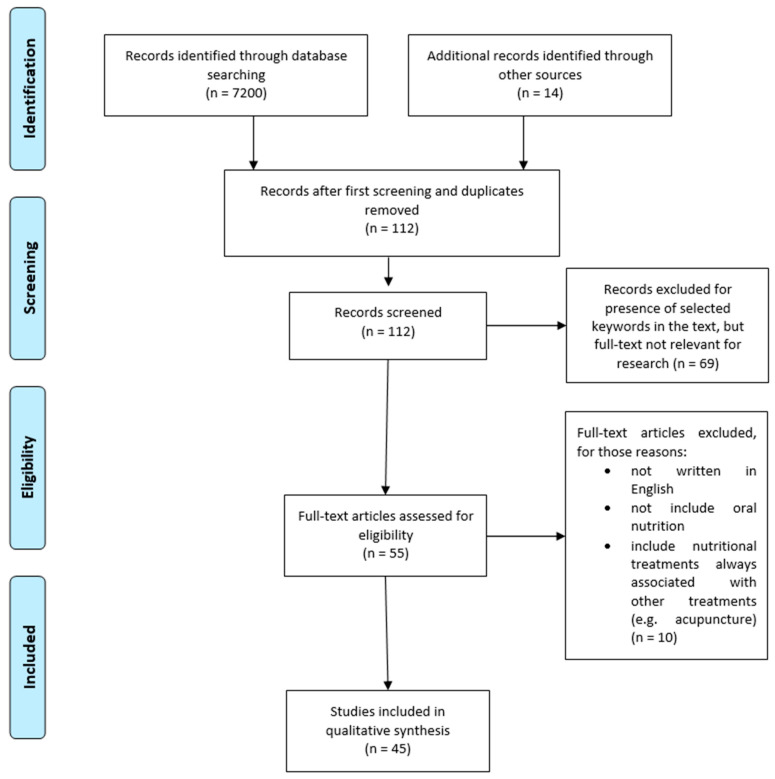
PRISMA flow diagram of studies selected.

The selection process adhered to the Preferred Reporting Items for Systematic Reviews and Meta-Analyses (PRISMA) guidelines [8], with each stage of the review documented in the PRISMA flow diagram (Figure 1). Articles were screened initially by title and abstract, followed by a full-text evaluation for inclusion based on the relevance and strength of evidence (following Oxford centre for Evidenze-Based Medicine, CEBM) [9].

For every study included, we evaluated the data summarized and the strength of evidence in order to create a hierarchy of evidence, which we subsequently compared against established nutritional guidelines for pregnancy (DGA, WHO, and FIGO nutritional checklist for food groups) in order to structure a food pyramid that addresses both the nutritional needs of pregnant women and the clinical management of NVP and HG.

## 3. Results

A total of 45 studies were included in the qualitative synthesis after the rigorous screening of 112 full-text articles, with exclusions primarily due to language limitations, the absence of oral nutrition interventions, or studies combining nutritional treatments with non-nutritional approaches. The included studies encompass randomized controlled trials, comparative studies, and systematic reviews, providing robust evidence for evaluating the efficacy of various nutritional strategies in managing nausea and vomiting during pregnancy (NVP).

In the following paragraphs, the evidence is analyzed by categorizing interventions into macronutrients, micronutrients, food groups, and botanical extracts of nutritional interest. This structured approach aims to highlight the specific therapeutic roles and clinical relevance of each category, providing a comprehensive understanding of their impact on NVP management.

### 3.1. Macronutrients

#### 3.1.1. Protein

The evidence in this section primarily includes observational studies, with a smaller number of interventional trials (one controlled study, two observational studies, one epidemiological study, one set of clinical guidelines, one prospective cohort study, one cross-sectional study, and one review). Observational data highlight consistent associations between higher protein intake and reduced NVP severity, while clinical trials provide additional support this by demonstrating the benefits of protein-rich meals in alleviating symptoms. The findings from these studies have been summarized in Table 1.

Jednak et al. evaluated the effects of protein-rich meals on nausea and gastric dysrhythmias among 14 pregnant women in their first trimester. Results demonstrated that protein meals significantly reduced both nausea and gastric dysrhythmic activity compared to carbohydrate and fat meals (*p* < 0.05). Electrogastrographic data indicated that while all nutrient types increased gastric electrical power, protein was uniquely effective in alleviating nausea symptoms without altering electrogastrographic power significantly, suggesting a distinct beneficial effect of protein on gastric motility regulation [10]. Latva-Pukkila et al. explored dietary intake variations in 134 pregnant women experiencing nausea and vomiting in pregnancy (NVP) compared to 53 women without symptoms. Findings showed that NVP was associated with a significantly lower protein intake (16.4 E%) versus non-NVP women (18.3 E%) (*p* = 0.003) and a higher carbohydrate proportion (*p* = 0.008). Such dietary alterations suggest potential impacts on maternal and fetal health due to nutrient imbalances induced by NVP [7]. Pepper and Roberts conducted a cross-cultural analysis, identifying a correlation between NVP prevalence and a higher intake of macronutrients, including protein, across various populations. However, restricting the analysis to North America and Europe revealed that macronutrient–NVP associations were not significant, implying possible geographical or lifestyle-related confounding factors [11]. Zhu et al. investigated a cohort of 303 Chinese women and found that those with NVP had notably lower intakes of protein, iron, and other essential nutrients compared to non-NVP women. Protein deficiency in particular was highlighted as a significant nutritional concern in women with severe NVP, correlating with a reduced gestational weight gain (*p* < 0.05), underlining the need for targeted nutritional interventions in this population [12]. Chortatos et al. conducted a large cohort study involving 51,675 Norwegian pregnancies and observed a significant relationship between nausea and vomiting in pregnancy (NVP) and dietary patterns. Protein intake was significantly lower in women with NVP compared to symptom-free women (mean intake: 87.3 g/day vs. 86.3 g/day, *p* < 0.001). Protein contributed to 15.3% of total energy in the NVP group, slightly lower than the 15.6% observed in the symptom-free group (*p* < 0.001). These results indicate a modest but statistically significant reduction in protein consumption in women experiencing NVP [6]. Cheng et al. examined dietary patterns among 3122 pregnant women and identified specific diet types associated with the risk of hyperemesis gravidarum (HG). Women with a diet rich in protein sources, such as eggs, milk, and seafood, had a 37–58% reduced risk of developing HG, while a high intake of sugary beverages increased the HG risk by 64% (*p*-trend < 0.05). These findings suggest a protective role of protein-rich foods against HG and highlight the potential adverse effects of high-sugar diets on severe pregnancy-related nausea [13]. Nelson-Piercy et al. discussed dietary recommendations in managing NVP and HG as part of comprehensive care. The guideline supports the use of dietary interventions, including increased protein intake, alongside antiemetic therapy. Specific proteins, such as thiamine-rich foods, are emphasized to prevent deficiencies that may exacerbate symptoms in women with severe vomiting [4]. Ballestín et al. provided a review highlighting the role of micronutrient supplementation, including protein sources, in addressing the nutrient deficiencies common in pregnancy. While the review does not focus solely on NVP, it discusses protein’s role in overall dietary quality for maternal and fetal health, particularly in countering nutritional inadequacies associated with nausea and vomiting [14].

In conclusion, the reviewed studies consistently highlight the beneficial role of protein intake in alleviating nausea and vomiting symptoms during pregnancy, especially in cases of severe nausea, such as hyperemesis gravidarum (HG). Protein-rich diets not only reduce gastric dysrhythmias but also address the nutritional inadequacies often seen in pregnant women with NVP, improving outcomes for maternal and fetal health [7,10,13]. Thus, incorporating protein sources into dietary recommendations may offer an effective, non-pharmacological approach to support maternal well-being and reduce the risks associated with nutrient deficiencies during pregnancy [4].

#### 3.1.2. Carbohydrate and Fiber

The evidence for carbohydrates and fiber is drawn mainly from observational cohort studies (two observational studies, one epidemiological study, one prospective cohort study). The role of carbohydrates, especially the distinction between simple and complex forms, appears to influence NVP symptoms significantly. The findings from these studies have been summarized in Table 2.

Latva-Pukkila et al. found that among pregnant women experiencing nausea and vomiting in pregnancy (NVP), the intake of simple sugars, as part of overall carbohydrate consumption, was significantly higher compared to non-NVP women (*p* = 0.008). This increase was primarily driven by a greater preference for foods rich in simple sugars, which may provide quick energy that could alleviate symptoms in the short term [7]. Pepper and Roberts analyzed cross-cultural data and found that populations with higher NVP rates consumed significantly more simple sugars, particularly from sugary beverages and snacks, while complex carbohydrate intake did not show a consistent correlation with NVP [11]. This finding suggests that simple sugars, rather than complex carbohydrates, might play a more direct role in either symptom relief or the prevalence of NVP in certain populations [7,11]. Chortatos et al. report that women with NVP consumed significantly more carbohydrates compared to symptom-free women (mean intake: 315.4 g/day vs. 301.9 g/day, *p* < 0.001). Carbohydrate intake accounted for 54.3% of total energy in the NVP group, compared to 53.5% in the symptom-free group (*p* < 0.001). Added sugar intake was also higher in the NVP group (65.5 g/day, 11.0% of energy) compared to the symptom-free group (59.8 g/day, 10.4% of energy, *p* < 0.001). However, while these differences were statistically significant, the variation in total carbohydrate intake is unlikely to hold clinical relevance. These results may primarily represent a statistical artifact rather than a meaningful association between carbohydrate intake and NVP symptoms. The study suggests that higher sugar intake might represent an attempt to mitigate NVP symptoms, but their role in symptom provocation cannot be excluded [6].

In conclusion, these studies highlight a distinct association between simple sugars and NVP, with women experiencing symptoms often consuming more simple sugars rather than complex carbohydrates. This suggests that dietary recommendations for NVP might benefit from managing simple sugar intake specifically, although further research is needed to determine whether this intake is primarily a response to symptoms or a potential factor in their exacerbation [6,7,11].

We identified only one study focusing on fiber intake in relation to nausea and vomiting in pregnancy (NVP), which nonetheless highlights its relevance in managing gastrointestinal symptoms associated with NVP and hyperemesis gravidarum. Reijonen et al. (2022) examined the relationship between dietary fiber intake and NVP, focusing on both soluble and insoluble fiber sources. Women with NVP were found to consume significantly more total fiber (*p* = 0.043) and specifically fiber from cereal products, representing mainly insoluble fiber, compared to women without NVP (*p* = 0.026). Despite higher intake levels, the study concluded that fiber did not appear to protect against NVP, although it was well-tolerated and maintained by women experiencing symptoms, suggesting that fiber may offer digestive benefits without influencing NVP onset [3].

In conclusion, this study implies that while dietary fiber intake, particularly insoluble fiber, does not prevent NVP, it remains well-tolerated during pregnancy and may aid in managing associated gastrointestinal symptoms. This suggests that fiber’s role could be supportive rather than preventative in NVP dietary recommendations [3].

#### 3.1.3. Lipids

An adequate intake of lipids, both in terms of quantity and quality, likely has a beneficial impact on the overall health of pregnant women. However, the results of our review did not identify any studies directly examining the relationship between the total intake of lipids, including saturated and unsaturated fats, and the occurrence of nausea, vomiting, or hyperemesis gravidarum during pregnancy. Findings from studies that linked lipid intake and NVP or hyperemesis gravidarum have been summarized in Table 3 (one observational study and one RCT).

Adu-Afarwuah et al. (2023) conducted a secondary analysis examining maternal morbidity, including nausea and vomiting, in pregnant women receiving different nutrient supplements. While lipids were part of the lipid-based nutrient supplements, no significant impact on the prevalence of gastrointestinal symptoms, including nausea and vomiting, was observed across groups, indicating that dietary lipids may not directly alleviate these symptoms [16]. Harding et al. (2017) analyzed the adherence to small-quantity lipid-based nutrient supplements (LNS) among 360 pregnant and lactating women enrolled in a cluster randomized trial in rural Bangladesh. Participants were provided one sachet of LNS daily, which included essential fatty acids and other nutrients, as part of a community health program. Adherence and acceptability were assessed through structured interviews and in-depth surveys. Reported vomiting at enrollment was negatively associated with adherence to LNS during pregnancy (OR: 0.34; 95% CI 0.14, 0.80; *p* = 0.014). Taste and smell aversions to LNS were frequently linked to nausea and vomiting, particularly during early pregnancy. Approximately 19% of women reported nausea as a reason for low adherence, with organoleptic properties, such as taste and smell, identified as barriers. Despite these challenges, women with high overall acceptability of the supplement reported significantly greater adherence (OR: 8.62; 95% CI 3.53, 20.83; *p* < 0.001). Programmatic factors, like regular health worker visits and uninterrupted supplement supplies, were critical to improving adherence [15].

In conclusion, no studies were identified that specifically analyzed the overall dietary intake of lipids in pregnant patients in relation to the risk of NVP. Nonetheless, existing literature suggests that moderate fat intake may provide a balanced approach for women experiencing NVP, whereas excessive fat consumption could potentially exacerbate symptoms [15,16].

### 3.2. Micronutrients

#### 3.2.1. Vitamins

The studies in this section include randomized controlled trials and observational analyses (four systematic reviews, four observational studies, two prospective studies, one retrospective analysis, eleven randomized controlled trials, two comparative studies, one experimental study, one clinical trial, and one matched cohort study). From the analysis of studies included in this review, evidence supports the role of multivitamins, vitamin D, B6, and B9 in reducing NVP symptoms. While vitamins B1, B2, B12, and C show fewer or no direct effects, their broader health benefits may indirectly support NVP management. Vitamins E and C’s antioxidant properties further contribute to maternal well-being. The findings from these studies have been summarized in Table 4 and Table 5.

**Vitamin E**: we reviewed multiple studies examining the effects of vitamin E (15 mg/day) on NVP. Although no direct impact on NVP symptoms was observed, the antioxidant properties of vitamin E may help mitigate oxidative stress linked to nausea and vomiting [19].

**Vitamin D**: a prospective study involving 1500 pregnant women to assess the relationship between vitamin D intake and NVP. Women with adequate serum 25(OH)D levels (≥50 nmol/L) had a 15% lower incidence of NVP compared to deficient women (*p* < 0.05). Similarly, Godfrey et al. [18] analyzed vitamin D levels in a cohort of 707 pregnant women, finding that adequate levels (≥30 ng/mL) correlated with reduced NVP symptoms. These results indicate the potential protective effects of vitamin D against NVP [17].

**Vitamin C**: Dror et al. found no direct effects of vitamin C (~85 mg/day) on NVP symptoms but noted that its antioxidant properties may help alleviate oxidative stress [19]. Foessleitner et al. conducted a clinical trial with 50 pregnant women using a vitamin C chewing gum (25 mg, four times daily), reporting a significant reduction in nausea intensity (*p* < 0.05). This suggests a potential supportive role for vitamin C in managing NVP [22].

**Vitamin B6**: vitamin B6 has been extensively studied for its antiemetic properties in the management of nausea and vomiting during pregnancy (NVP). A systematic review and meta-analysis by Jayawardena et al. (2023), which included 18 studies, highlighted the consistent efficacy of vitamin B6 in reducing both the severity and frequency of nausea. The pooled effect size for nausea reduction according to the Rhode’s score was 0.78 (95% CI: 0.26, 1.31; *p* = 0.003), and for the PUQE score, it was 0.75 (95% CI: 0.28, 1.22; *p* = 0.002). The review also emphasized the significant improvement observed in combination therapies, particularly with doxylamine. [24]. A systematic review and meta-analysis by Gaur et al. (2022) included seven RCTs with 819 participants comparing ginger and vitamin B6 for the management of NVP. The review found that vitamin B6 significantly improved overall NVP scores compared to ginger (SMD 0.36, 95% CI 0.06 to 0.65; *p* = 0.02). However, no significant differences were noted for reducing nausea severity (*p* = 0.14) or the vomiting frequency (*p* = 0.57), reinforcing vitamin B6’s role in overall symptom management [23]. A triple-blind randomized trial by Sharifzadeh et al. (2018) evaluated vitamin B6 (40 mg twice daily), ginger, and a placebo in 77 pregnant women with NVP. The study found that vitamin B6 reduced nausea by 60% compared to the placebo (*p* < 0.01), demonstrating its efficacy for mild-to-moderate symptoms [29]. Similarly, Ensiyeh and Sakineh (2009) conducted a randomized controlled trial comparing vitamin B6 (40 mg/day) to ginger (1 g/day) in 70 pregnant women under 16 weeks of gestation. Both treatments reduced nausea by 55% (*p* < 0.01), with no significant difference between the groups (*p* = 0.85), suggesting comparable effectiveness [25]. Firouzbakht et al. (2014) investigated the efficacy of vitamin B6 (40 mg twice daily) versus ginger (500 mg twice daily) in 80 pregnant women over one week. Both interventions reduced nausea severity by 50% (*p* = 0.03), with no significant difference in outcomes but no statistical significance (*p* = 0.7) [26]. Haji Seid Javadi et al. (2013) compared vitamin B6 (30 mg twice daily) with ginger (1 g/day) in 90 pregnant women and found that vitamin B6 led to a 70% reduction in nausea, slightly outperforming ginger’s 60% reduction (*p* < 0.05). Both treatments were well-tolerated and safe [27]. Pope et al. (2015) conducted a matched cohort study with 160 pregnant women, evaluating pyridoxine (10 mg/day) against a doxylamine-pyridoxine combination. The combination therapy achieved a 75% reduction in PUQE scores, significantly outperforming pyridoxine alone, which showed a 50% reduction (*p* < 0.01). This highlights its higher efficacy, particularly in severe cases [28]. Wibowo et al. (2012) assessed vitamin B6 supplementation (10 mg/day) in 60 pregnant women with NVP over two weeks. The study reported a 45% reduction in nausea and a 40% reduction in vomiting (*p* < 0.05), with good tolerability among patients [30]. A randomized controlled trial by Vutyavanich et al. (1995) involving 342 women found that vitamin B6 (30 mg/day) significantly reduced nausea severity compared to a placebo (*p* = 0.0008), though the effect on vomiting episodes did not reach statistical significance (*p* = 0.0552) [33]. Similarly, Koren et al. (2010) demonstrated a significant improvement in PUQE scores with doxylamine-pyridoxine (Diclectin) compared to the placebo (−4.8 vs. −3.9, *p* = 0.006), highlighting its efficacy in mild-to-moderate NVP cases [34]. Tan et al. (2009) evaluated pyridoxine (20 mg thrice daily) in women with severe NVP or hyperemesis gravidarum (HG) and found no significant differences in symptom improvement compared to the placebo (*p* > 0.05), suggesting limited efficacy for severe cases [35]. Persaud et al. (2018) reported modest symptom improvements with doxylamine-pyridoxine, but the clinical relevance of these results remains debated [36].

**Vitamin B1 (Thiamine)**: a retrospective analysis was conducted on 1500 pregnant women to evaluate the effects of multivitamins containing thiamine (1.4 mg/day) on NVP symptoms. While there was a mild reduction in NVP severity among women taking thiamine, the effect was not statistically significant (*p* = 0.08). This suggests that thiamine’s direct impact on NVP is limited but may still provide some support within a multivitamin regimen [20].

**Vitamin B12**: multiple studies were reviewed focusing on vitamin B12 and its association with NVP. They reported that low B12 levels (<200 pg/mL) were significantly linked to increased NVP severity. Adequate intake (~2.6 mcg/day) was recommended for neurological and overall maternal health, which may indirectly alleviate NVP symptoms. Godfrey et al. conducted an observational study involving 707 pregnant women to assess the relationship between B12 levels and NVP severity [18]. Women with B12 deficiency (<200 pg/mL) experienced more severe NVP symptoms compared to those with sufficient levels, emphasizing the importance of maintaining adequate B12 intake during pregnancy [19].

**Folate (Vitamin B9)**: we found an investigation of the effects of folic acid (400 mcg/day) within a multivitamin in 1046 pregnant women. The study found a 12% reduction in NVP incidence among women taking folic acid compared to those not supplemented (*p* = 0.02). These results support folate’s role in reducing the risk and severity of nausea during pregnancy [21].

**Riboflavin (Vitamin B2)**: one study analyzed the effects of riboflavin intake as part of a standard prenatal multivitamin in 707 pregnant women. While no direct effects on NVP symptoms were observed, riboflavin was noted to support overall metabolic health, which may indirectly benefit pregnant women experiencing nausea [18].

**Multivitamins:** a randomized controlled trial conducted with 1046 pregnant women to evaluate the effects of multivitamins on NVP. Participants taking a multivitamin containing vitamins B, C, D, and E reported a 12% reduction in NVP incidence compared to the non-supplemented group (*p* = 0.02). These findings support the protective role of multivitamins in NVP management [21].

In conclusion, evidence strongly supports the role of vitamin B6 as a highly effective and well-tolerated treatment for nausea and vomiting in pregnancy (NVP), with consistent reductions in symptom severity across multiple studies. While vitamins B1, B2, B12, and B9 show fewer direct effects on NVP, they contribute to overall maternal health and may indirectly alleviate symptoms. Vitamin B12 deficiency is notably linked to increased NVP severity, underscoring the need for adequate intake. Collectively, the B-vitamin group plays a pivotal role in managing NVP, with vitamin B6 standing out as the most impactful intervention.

#### 3.2.2. Minerals

The studies in this section include randomized controlled trials and observational analyses (four randomized controlled trials, four observational studies, and one randomized trial). Current evidence suggests that certain minerals, notably zinc, potassium, and selenium, may play a role in managing nausea and vomiting in pregnancy (NVP), with zinc and potassium potentially associated with reduced symptom severity. Iron, calcium, and phosphorus contribute to maternal health without directly alleviating NVP, while selenium intake has been correlated with NVP severity. These findings indicate a nuanced role for mineral intake, where certain nutrients might indirectly support NVP management through overall maternal health. The findings from these studies have been summarized in Table 6.

**Zinc**: zinc supplementation has shown potential in reducing pregnancy-related complications, which may indirectly influence NVP severity. Nossier et al. (2015) conducted a randomized controlled trial among 675 pregnant women with low baseline zinc levels, showing that 30 mg/day of zinc sulfate (ZnSO4) significantly reduced pregnancy complications and early neonatal infections (*p* < 0.05). Although a direct effect on NVP was not reported, these outcomes suggest zinc’s role in immune support may indirectly benefit women with NVP [37]. Additionally, Zhu et al. (2023) found a significant association between low dietary zinc intake and increased NVP severity among 303 Chinese women (*p* < 0.05), suggesting a possible relationship between zinc deficiency and worsening NVP symptoms [12].

**Potassium**: potassium intake may influence NVP symptoms by helping maintain the electrolyte balance. Zhu et al. (2023) reported significantly lower potassium intake among pregnant women with NVP compared to those without (*p* < 0.05). Reduced potassium intake may exacerbate electrolyte imbalances, which are common in NVP and contribute to symptoms like fatigue and nausea. Although causation is not established, adequate potassium intake might support NVP symptom management by stabilizing electrolyte levels [12].

**Iron**: while iron supplementation supports maternal health, its impact on NVP remains indirect. Bhutta et al. (2009) conducted a randomized controlled trial comparing iron and folic acid supplementation to a multivitamin containing iron, finding a significant reduction in anemia and iron deficiency in the iron-supplemented group (*p* < 0.05). However, no specific decrease in NVP symptoms was noted, indicating that iron primarily benefits general maternal health rather than directly alleviating NVP [38]. Zhou et al. (2009) performed a dose-response trial, comparing 20, 40, and 80 mg daily doses of iron among anemic pregnant women. Results showed that the 20 mg dose effectively improved hemoglobin levels with fewer gastrointestinal side effects, suggesting that lower doses may reduce NVP-related discomfort linked to iron supplementation (*p* < 0.05) [39].

**Calcium**: calcium is crucial for maternal bone health and fetal development, though its effect on NVP is unclear. Adu-Afarwuah et al. (2023) studied calcium intake among 500 Ghanaian pregnant women, finding that daily calcium supplementation improved the maternal bone density and fetal growth (*p* < 0.05). Despite the lack of direct impact on NVP symptoms, calcium’s support for maternal health may indirectly assist women managing NVP by promoting baseline well-being [16].

**Selenium**: Selenium intake has been observed to correlate with NVP severity. Zhu et al. (2023) reported lower selenium intake among pregnant women experiencing NVP (*p* < 0.05), suggesting that selenium deficiency might be linked to worsened NVP symptoms. This association may highlight selenium’s role in antioxidant defense, which could potentially aid in reducing the oxidative stress that can exacerbate NVP symptoms, although the direct effects on symptom relief are yet to be determined [12].

**Phosphorus**: phosphorus intake may also relate to NVP severity, though evidence is limited. Zhu et al. (2023) found that phosphorus intake was lower among women with NVP (*p* < 0.05), potentially due to nutritional imbalances caused by dietary changes related to NVP. This suggests that phosphorus might indirectly support NVP symptom management through its role in energy metabolism, although further studies are required to clarify this link [12].

In summary, zinc, potassium, and selenium show the strongest associations with NVP, where the adequate intake of these minerals may provide indirect symptom relief. Zinc and potassium appear inversely related to the NVP prevalence, and selenium’s role in antioxidant support may offer additional benefits. While iron, calcium, and phosphorus primarily support general maternal health, they do not directly influence NVP. Further research is warranted to clarify the roles of these minerals in NVP management and to guide dietary recommendations for pregnant women.

### 3.3. Food Groups and Botanical Extracts of Nutritional Interest

The studies reviewed for food groups are predominantly observational, focusing on dietary patterns involving fruits, vegetables, and cereals (three observational studies, two cohort studies, and three validation studies). Current evidence suggests that dietary patterns involving specific food groups, particularly high-protein foods, fruits and vegetables, and fiber-rich cereals, may help alleviate the symptoms of nausea and vomiting in pregnancy (NVP). Conversely, dietary patterns high in sugars and certain beverages may exacerbate these symptoms. Here, we examine the impact of different food groups on NVP based on studies exploring meat, fish, eggs, fruits, vegetables, and cereals. The findings from studies regarding food groups have been summarized in Table 6 and the findings from studies regarding botanical extracts are summarized in Table 7 and Table 8.

**Meat, Fish, and Eggs**: high-protein foods, including meat, fish, and eggs, have been linked to a lower incidence of NVP. Cheng et al. observed a 37% reduction in the risk of hyperemesis gravidarum (HG) among women who consumed these foods regularly, particularly with 3–4 servings of fish per week (*p*-trend < 0.05), suggesting a protective effect of protein-rich foods on severe NVP symptoms [13]. Latva-Pukkila et al. further found that women with NVP consumed fewer servings of meat and derived a smaller proportion of their daily caloric intake from animal protein (*p* = 0.007), indicating that reduced meat consumption may correlate with symptom management [7]. Ogawa et al. conducted a study among Japanese pregnant women, noting that those with lower NVP symptoms had higher intake of eggs and fish (2–3 times per week) compared to those with NVP (*p* < 0.05), suggesting a potential benefit of including these foods in dietary recommendations [40].

**Fruits and Vegetables**: fruits and vegetables have been consistently associated with reduced NVP symptoms. Vézina-Im et al. validated a brief questionnaire and found that a high intake of fruits and vegetables (at least five servings per day) correlated with lower nausea intensity, attributed to their fiber content, hydration benefits, and array of vitamins that support digestive health [41]. Ogawa et al. found similar results among Japanese pregnant women, where higher vegetable intake (3–5 servings per week) was linked to fewer NVP symptoms (*p* < 0.05), reinforcing the role of a fruit- and vegetable-rich diet in managing NVP symptoms [40].

**Cereals and Fiber-Rich Foods**: fiber from grains and cereals appears to positively impact NVP management. Reijonen et al. observed that women who consumed an average of 3–4 servings of fiber-rich foods daily maintained their intake even when experiencing NVP, suggesting that fiber is well-tolerated and may help to mitigate symptoms [3]. Zhu et al. noted that lower fiber intake was associated with more severe NVP symptoms (*p* < 0.05) [12], while Ogawa et al. reported that higher cereal intake (2–3 servings daily) was linked to a reduced NVP incidence among non-NVP participants (*p* < 0.05), suggesting that whole grains and fiber-rich foods stabilize blood glucose and support digestive health in pregnancy [40].

Overall, the reviewed studies indicate that specific dietary patterns, particularly those high in lean proteins, fruits, vegetables, and fiber-rich cereals, may provide symptomatic relief for NVP. High-fiber diets, along with lean protein and hydration-supportive fruits and vegetables, offer potential dietary interventions to alleviate nausea and stabilize digestive health during pregnancy. However, high sugar and certain beverages may exacerbate symptoms, underscoring the importance of balanced dietary patterns in NVP management. Further research is warranted to explore these associations in more depth and provide targeted dietary recommendations for pregnant women [3,7,12,13,41].

**Botanical extract**: In the process of selecting botanical extracts, only those with nutritional relevance and intended for ingestion, rather than aromatherapy, were considered. Consequently, ginger emerged as the sole botanical of interest, given its established antiemetic effects and common use in dietary supplements to manage nausea and vomiting in pregnancy (NVP). The evidence for ginger is robust, with several randomized controlled trials and meta-analyses demonstrating its efficacy in reducing nausea and vomiting symptoms (nine randomized controlled trials, three comparative studies, one experimental study, one systematic review and meta-analysis, and one case-control study). Ginger has emerged as a prominent botanical extract for its antiemetic effects in the management of NVP. A systematic review and meta-analysis by Hu et al. (2020), which analyzed data from 1174 participants across 13 randomized controlled trials, demonstrated a significant pooled effect size of 0.821 (95% CI: 0.585, 1.056; *p* < 0.001) for reducing nausea intensity. [42]. The systematic review by Gaur et al. analyzed 7 RCTs with 819 participants and found that ginger’s efficacy was comparable to vitamin B6 in alleviating nausea severity (*p* = 0.14) and vomiting frequency (*p* = 0.57) but with non-statistical significance. Despite these findings, vitamin B6 was more effective in improving overall NVP scores (SMD 0.36, 95% CI 0.06 to 0.65; *p* = 0.02), positioning ginger as a viable alternative for mild-to-moderate cases of NVP [23]. The meta-analysis also highlighted consistent efficacy in reducing both the severity and frequency of vomiting, with a high safety profile and minimal adverse effects. The authors emphasized the need for standardized dosing protocols to improve reproducibility and ensure consistent therapeutic outcomes [23]. Ansari et al. (2016) conducted a randomized controlled trial involving 80 breast cancer patients undergoing chemotherapy, using ginger extract (1 g/day) compared to placebo over five days. The ginger group experienced a 60% reduction in nausea scores, significantly higher than the placebo group’s 30% reduction (*p* < 0.05). Vomiting episodes also decreased by 55% in the ginger group (*p* < 0.05), supporting its effectiveness as an antiemetic [43]. In a comparative study, Asatova et al. (2019) evaluated ginger (1 g/day) against vitamin B6 (40 mg/day) in 100 pregnant women with moderate nausea. Both groups demonstrated a 55% improvement in symptom severity, with no significant difference in outcomes (*p* = 0.7), suggesting similar efficacy for mild-to-moderate cases [31]. Basirat et al. assessed ginger biscuits (250 mg per biscuit, twice daily) in 60 pregnant women during the first trimester. The intervention resulted in a 50% reduction in nausea (*p* < 0.01) and a 45% decrease in the vomiting frequency (*p* < 0.01), with excellent patient tolerance and no adverse effects [44]. Fischer-Rasmussen et al. conducted a randomized controlled trial involving 70 women with hyperemesis gravidarum, comparing ginger capsules (250 mg, four times daily) with a placebo over four days. Ginger led to a 70% reduction in nausea and a 60% reduction in the vomiting frequency (*p* < 0.01), outperforming the placebo and demonstrating its potential for severe NVP cases [45]. Pongrojpaw et al. compared ginger (500 mg twice daily) with dimenhydrinate (50 mg twice daily) in 120 pregnant women. Both treatments showed similar reductions in nausea (65% for ginger vs. 68% for dimenhydrinate, *p* = 0.65), with ginger showing fewer side effects, such as drowsiness, making it a preferable alternative [46]. Smith et al. compared ginger (1.05 g/day) with vitamin B6 (75 mg/day) in a randomized controlled trial involving 291 pregnant women. Both groups showed a 60% reduction in symptoms (*p* = 0.8), with high tolerability and no reported adverse effects. This study supports ginger’s safety and effectiveness as comparable to vitamin B6 for early pregnancy management [32]. A randomized controlled trial by Saberi et al. involving 120 pregnant women found that ginger (500 mg/day) significantly reduced nausea and vomiting compared to the placebo (*p* < 0.001) [47]. Similarly, Ozgoli et al. (2009) demonstrated significant reductions in nausea severity with ginger capsules (1 g/day) compared to the placebo (*p* < 0.001), confirming its effectiveness in moderate cases [49]. Rukh et al. evaluated the use of “Gingocap” ginger capsules compared to pyridoxine (30 mg/day) over 8 weeks and found greater symptom relief with ginger (*p* = 0.03), along with high patient satisfaction and minimal side effects [48].

In summary, these studies consistently demonstrate that ginger is an effective and safe option for reducing nausea and vomiting in pregnancy, showing comparable efficacy to conventional antiemetics, such as vitamin B6 and dimenhydrinate. The consistent reductions in nausea and vomiting across various dosages and forms, such as ginger capsules and biscuits, support ginger’s inclusion in dietary recommendations for managing NVP.

## 4. Discussion

Nausea and vomiting in pregnancy (NVP) require a targeted dietary strategy to alleviate symptoms while meeting maternal and fetal nutritional requirements. From the findings selected from studies included in this review, several practical recommendations emerge, suggesting minor but impactful modifications to current dietary practices. These adjustments align closely with recognized nutritional standards for pregnant women, ensuring symptom management without compromising maternal and fetal health.

Consuming 45–60% of total energy from carbohydrate intake is essential for stabilizing blood glucose levels and reducing NVP severity [7]. We suggest that preferring complex carbohydrates, such as whole grains, legumes, and starchy vegetables, aligns with LARN, WHO, and DGA guidelines. However, simple sugars, while sometimes better tolerated during acute symptoms, should remain below 10% of total energy to prevent glycemic instability and symptom exacerbation. Complex carbohydrates also provide slower glucose release, minimizing the risk of reactive hypoglycemia—a common trigger for nausea in early pregnancy [6,7,11].

**Figure 2:**
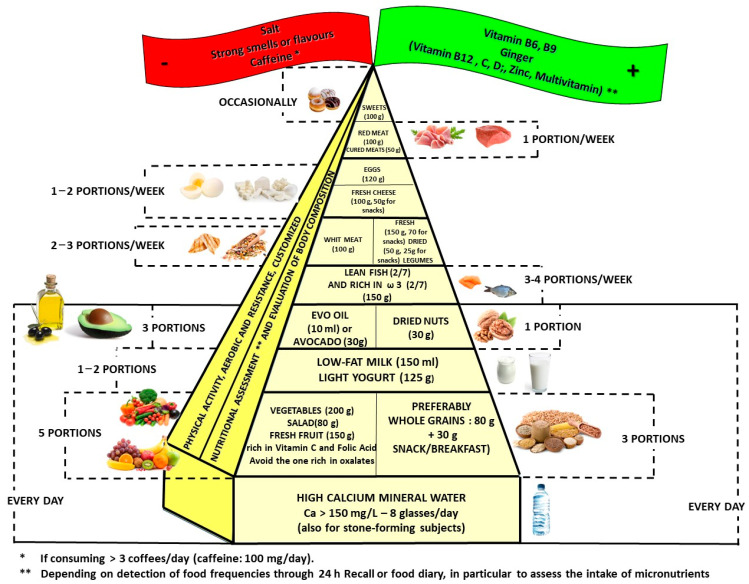
Nutritional pyramid of the prevention and management of NVP and HG.

Protein intake, as outlined by official guidelines (WHO: 10–15% of energy, DGA: 10–35% of energy), is sufficient for NVP and HG management. However, evidence suggests that dividing protein intake across five meals throughout the day improves gastric motility and reduces nausea intensity by sustaining amino acid availability and preventing gastric dysrhythmias [10]. We also suggest emphasizing the consumption of lean protein sources, such as poultry (2–4 servings per week), fish (3–4 servings per week), eggs (1–2 servings per week), and legumes (2–4 servings per week), which are well-tolerated and align with recommendations for maintaining a balanced nitrogen balance in pregnancy [7,13,40], with portion sizes depending on the nutritional needs and meal type (eg. snacks, or main meals, like breakfast, lunch, or dinner), as reported in Figure 2. In conclusion, an important factor in the dietary control of nausea and NVP is not only the frequency of protein sources but also the distribution of protein intake among many meals and snacks throughout the day. Evidence suggests that fractioning protein consumption enhances gastric motility and reduces nausea intensity by maintaining a steady supply of amino acids, which may help mitigate gastric dysrhythmias [10]. This strategy aligns with current recommendations that emphasize small, frequent meals to improve symptom tolerance [4]. Most importantly, snacks should also include a source of protein (including nuts) to optimize nutritional intake and symptom relief. Preferred protein options include yogurt or milk (preferably low-fat), a boiled egg—while monitoring daily cholesterol intake to maintain overall dietary balance—and legumes, which serve as a versatile, plant-based protein alternative [7,40]. Incorporating such protein-rich snacks not only improves gastric stability but also provides practical solutions to address the nutrient deficiencies commonly associated with severe NVP [12].

Fats should account for 20–35% of total energy intake, focusing on unsaturated sources, like olive oil, nuts, seeds, and avocados. Excessive saturated fat intake slows gastric emptying, aggravating nausea and necessitating limits below 10% of energy, as stipulated by all major guidelines [10]. Furthermore, the inclusion of essential fatty acids supports anti-inflammatory pathways, potentially mitigating the oxidative stress associated with NVP [19]. From the selected studies, there is no specific evidence supporting the use of specific lipid products (es avocado, extra virgin olive oil, EVOO). Therefore, any fat sources rich in unsaturated fats, such as avocado and seeds, are recommended. Nevertheless, it is possible that some of these, such as EVOO, might offer superior benefits compared to others.

Vitamin B6 remains a cornerstone intervention for managing NVP, with therapeutic doses ranging from 30 to 40 mg/day emerging as the standard for symptom alleviation during pregnancy. These doses significantly exceed the baseline dietary recommendations (1.3–1.7 mg/day), emphasizing the necessity of targeted supplementation. Combination therapies, particularly with doxylamine, have shown enhanced benefits, especially in severe cases, highlighting their clinical importance in specific populations. The biomolecular action of vitamin B6 involves neurotransmitter synthesis, including serotonin and gamma-aminobutyric acid (GABA), as well as hormonal modulation, which may alleviate nausea and vomiting [23,24,42].

Ginger extract is another effective intervention for NVP, with therapeutic doses ranging from 500 mg to 1 g/day. Its bioactive compounds, such as gingerols and shogaols, exert anti-inflammatory and prokinetic effects and act as serotonin receptor antagonists within the gastrointestinal tract, contributing to its antiemetic properties. These mechanisms make ginger a viable alternative or adjunct to vitamin B6 for patients who cannot tolerate pharmacological treatments [23,42]. Ginger’s ability to modulate gastrointestinal motility and reduce the activation of nausea-inducing pathways highlights its potential use as part of an integrated approach to managing NVP. Additionally, its minimal adverse effects and widespread availability make it a practical choice for many pregnant women, especially those seeking non-pharmacological solutions [23,42].

Zinc (30 mg/day) supplementation has been linked to reductions in oxidative stress and improved NVP outcomes, especially in women with lower dietary zinc intake [12]. Vitamin B12 (2.4 µg/day) deficiencies have been associated with increased NVP severity, underscoring the need for adequate intake to maintain maternal neurological function [18,19]. Vitamin C (~85 mg/day) does not directly alleviate NVP but provides antioxidant support to reduce oxidative stress linked to nausea [22]. Vitamin D (15 µg/day) reduces NVP prevalence by addressing deficiencies that exacerbate symptoms [17]. Selenium (60–70 µg/day) alleviates oxidative stress and inflammation, both of which are implicated in the pathophysiology of NVP and HG [37].

Folate (vitamin B9), while not directly effective in managing NVP or HG, is indispensable in pregnancy for preventing neural tube defects and supporting fetal neurological development. Given that this dietary pyramid is designed for pregnant women, folate supplementation (4–5 mg/day) has been included in the green banner to align with official prenatal care recommendations [21]. Its inclusion ensures comprehensive nutritional support for maternal and fetal health.

Another important consideration is the preparation and sensory appeal of food, as certain odors and tastes can exacerbate nausea. Evidence suggests that cooking in well-ventilated areas or preparing minimally seasoned meals may help women with increased olfactory sensitivity [45]. While the official guidelines rarely address food preparation in the context of NVP, practical adjustments—such as avoiding fried or heavily spiced dishes—can improve food tolerance and adherence to nutritional recommendations. Cold or room-temperature foods, such as salads, smoothies, or cold protein sources (e.g., boiled eggs, cottage cheese), are often better tolerated than hot dishes, which produce stronger aromas that may trigger nausea [46]. Incorporating such alternatives not only enhances dietary compliance but also fills a gap in standard guideline recommendations for managing NVP and HG through practical culinary adjustments.

The dietary pyramid also highlights the importance of both including and avoiding specific substances, as represented in the green and red banners, respectively.

The green banner emphasizes the importance of supplementing with vitamin B6, ginger, vitamin B12, zinc, vitamin C, selenium, and vitamin D, as well as folate. These nutrients have demonstrated either direct efficacy in managing NVP and HG or essential roles in supporting overall maternal health during pregnancy. However, the nutrients included in parentheses in the green banner (vitamin B12, vitamin C, selenium, and vitamin D) are considered optional supplements. This is because the available evidence supporting their use is less robust, derived from lower levels of evidence, or lacks clarity regarding whether the observed benefits apply to women with marginal deficiencies or those with adequate baseline levels. Additionally, the recommended doses for these nutrients can typically be met through a balanced diet, and supplementation may only be necessary in cases of confirmed deficiencies or significant dietary restrictions.

The red banner highlights the need to limit or avoid caffeine, strong smells, and high-sodium foods. Caffeine, though not directly linked to worsening NVP or HG, should be limited to 200 mg/day (approximately one to two cups of coffee) due to its association with adverse pregnancy outcomes, including an increased miscarriage risk and reduced fetal growth at high intake levels [50]. Strong smells and heavily seasoned foods can trigger nausea in women with heightened olfactory sensitivity, a common physiological change during pregnancy. Cooking in well-ventilated areas, avoiding fried or spicy dishes, and opting for cold or room-temperature foods can mitigate these triggers [45,46]. High-sodium foods, while not directly exacerbating NVP, can worsen fluid retention and hypertension, indirectly affecting maternal well-being. Limiting sodium intake to below 1 g/day supports cardiovascular health and helps to manage overall pregnancy-related discomfort [51].

### 4.1. Elaboration of the Nutritional Pyramid

The pyramid tailored for managing nausea and vomiting in pregnancy (NVP) is illustrated in Figure 2. Additionally, Supplementary Materials provides an example of a 2000 kcal daily meal plan that incorporates the recommended portion sizes outlined in the pyramid in Figure 2, offering a practical application of these guidelines.

To contextualize the dietary strategy within broader nutritional standards, we have included Table 9 and Table 10, which compare the nutritional goals for pregnant women as defined by major international guidelines and the FIGO Nutrition Checklist. These tables serve as a reference for recommended portion sizes across various food groups, forming the foundation for meal planning that aligns with both general pregnancy requirements and the specific needs of women experiencing NVP.

The proposed pyramid for managing NVP differs from general pregnancy nutrition guidelines in several key aspects to better address symptom management and cultural adaptability. One notable difference is the inclusion of protein sources, such as legumes, not only in main meals but also in snacks. This approach diversifies protein intake beyond traditional options, like yogurt and milk, accommodating individuals in cultural contexts (e.g., China) where dairy consumption is less common. Additionally, the pyramid avoids an overemphasis on foods with strong odors, such as red meat and eggs, which could exacerbate symptoms for women with increased olfactory sensitivity. This aspect was carefully considered to balance nutritional adequacy with symptom management, as such foods might be poorly tolerated by individuals with heightened olfactory sensitivity during pregnancy. Future research could further explore the nutritional implications of this relationship and identify suitable alternatives to meet protein requirements without triggering discomfort.

If deemed appropriate by the clinician, other botanicals with evidence for general nausea and vomiting, such as those discussed in Giacosa et al. could be considered, despite the lack of specific evidence for NVP and HG [53]. However, it is recommended to prioritize those included in this pyramid, given their specific evidence of efficacy in managing NVP and HG symptoms.

Another distinctive feature is the increased recommendation for legumes, which serve as a versatile protein source in meals and snacks while minimizing reliance on animal-based proteins. This adaptation ensures a balanced intake without contributing to nausea triggered by specific food smells. The pyramid also incorporates global cultural preferences and dietary practices, offering flexibility in food group selection to support adherence in diverse populations. These adjustments make the pyramid a practical tool for symptom relief while aligning with the overall nutritional needs of pregnant women across different regions and dietary habits.

### 4.2. Future Implications

The effective dietary management of nausea and vomiting in pregnancy (NVP) and hyperemesis gravidarum (HG) through structured nutritional strategies, such as the proposed food pyramid, could lead to significant clinical and economic benefits. By reducing the severity of symptoms, this approach may decrease the reliance on pharmacological treatments and hospitalizations, thereby minimizing potential side effects, healthcare costs, and the burden on medical systems. For example, fewer interventions might translate into better resource allocation in healthcare settings, particularly in regions where access to prenatal care is limited.

Moreover, improved symptom control can enhance the quality of life for pregnant women, fostering better emotional well-being and reducing stress-related complications. Optimized nutrient intake through tailored dietary strategies also supports fetal development, potentially reducing the risk of low birth weights and other complications associated with severe maternal malnutrition. On a broader scale, these findings could inform public health initiatives aimed at creating accessible dietary guidelines for pregnant populations, integrating both cultural and regional dietary preferences. By emphasizing proactive nutritional management, this approach underscores the importance of personalized care in promoting healthier pregnancy outcomes for both mothers and their babies.

### 4.3. Limitations of the Study

This study has several limitations. First, the exclusion of studies not directly addressing dietary strategies for managing NVP may have resulted in the omission of potentially relevant but indirectly related evidence. Second, the reliance on keywords during the search strategy may have limited the scope of included studies, excluding some that could contribute additional insights. Third, the heterogeneity of study designs and methodologies among the included articles posed challenges in synthesizing the evidence and drawing consistent conclusions. Fourth, the lack of standardized dosing protocols for certain dietary interventions, such as ginger or vitamin B6, limits the generalizability of the findings to broader populations. Additionally, the absence of long-term follow-up data from the included studies prevents an evaluation of sustained dietary effects on NVP management. Finally, cultural and regional dietary differences may influence the applicability of the proposed recommendations, underscoring the need for localized research to refine dietary guidelines. Future research should aim to address these gaps to strengthen the evidence base.

## 5. Conclusions

Nausea and vomiting in pregnancy (NVP) represent significant challenges for maternal well-being, requiring a dietary strategy that not only alleviates symptoms but also ensures maternal and fetal nutritional adequacy. The dietary pyramid proposed for managing NVP, illustrated above, integrates evidence-based guidelines and practical recommendations to achieve these goals while addressing cultural and individual dietary needs.

This pyramid emphasizes a balanced distribution of macronutrients. Complex carbohydrates should account for 45–60% of total energy intake, sourced from whole grains, legumes, and starchy vegetables, as they stabilize blood glucose levels and reduce nausea severity. Simple sugars, although sometimes better tolerated during acute symptoms, should remain below 10% of total energy to avoid glycemic instability and symptom exacerbation. Protein intake, representing 10–35% of total energy, is optimized by distributing it across five meals daily. This approach improves gastric motility, reduces nausea intensity, and sustains amino acid availability. Recommended protein sources include lean white meats (100 g, 2–4 portions/week), fish (150 g, 3–4 portions/week), eggs (60 g, 1–2 portions/week), and legumes (50 g dried, 2–3 portions/week). These options are nutrient-dense and well-tolerated, accommodating cultural preferences and individual tolerances.

Fats should contribute 20–35% of total energy, focusing on unsaturated sources, like extra virgin olive oil (10 mL/day), nuts (30 g, 1 portion/week), seeds, and avocados, while saturated fats should remain below 10% of energy to avoid delayed gastric emptying and aggravation of symptoms. Essential fatty acids, particularly omega-3s from fish, support anti-inflammatory pathways, which may alleviate the oxidative stress associated with NVP.

Micronutrient supplementation plays a critical role in NVP management. Vitamin B6, at therapeutic doses of 30–40 mg/day, has consistently demonstrated effectiveness in reducing symptoms. Folate (4–5 mg/day), although not directly beneficial for NVP management, is essential for preventing neural tube defects and supporting fetal neurological development. Similarly, vitamins B12 (2.4 µg/day), C, and D (15 µg/day) are vital for maternal and fetal health, with vitamin D supplementation shown to reduce the NVP prevalence. Minerals, such as zinc (30 mg/day), potassium, and selenium, further alleviate symptoms by improving the electrolyte balance and reducing oxidative stress.

Foods with strong odors should be minimized across all food groups, including fish, white meat, and vegetables, as they can exacerbate nausea in women with increased olfactory sensitivity. This recommendation is distinct from portion guidelines, which aim to meet nutritional needs and ensure dietary adequacy. For example, red meats (100 g, 1 portion/week) and cured meats (50 g) are included in limited amounts primarily to mitigate the risks of cardiovascular disease and cancer associated with excessive consumption, rather than due to their odors. By separating these considerations, the pyramid ensures clarity in addressing both nutritional adequacy and practical management of nausea triggers.

Additionally, sodium intake should be limited to 1 g/day and caffeine to 200 mg/day (approximately 1–2 cups of tea or coffee), to minimize the potential exacerbation of symptoms. The pyramid’s flexibility and cultural adaptability make it a practical tool for symptom relief. For instance, the inclusion of legumes as a versatile protein source addresses dietary patterns in populations where dairy consumption is less common. The daily and weekly portion recommendations align with major guidelines, such as WHO and DGA, ensuring nutritional adequacy while alleviating NVP symptoms. This approach provides a structured yet adaptable framework to manage NVP effectively, supporting maternal and fetal health across diverse populations. Future research should explore further cultural adaptations and evaluate the long-term benefits of this dietary strategy in managing NVP and ensuring optimal pregnancy outcomes.

## Figures and Tables

**Table 1:** Studies reporting data regarding protein intake and NVP or HG.

Author and Year	Study Type (Evidence Strength)	Methods	Dosage	Results and Conclusions
[10]	Controlled Study (Level II)	Controlled study with 14 pregnant women experiencing first-trimester nausea. Compared protein-predominant meals with carbohydrate and fat meals for nausea relief, using electrogastrography to measure gastric dysrhythmias.	Participants consumed three meals per day: protein-predominant meals (30% protein, 20% fat, 50% carbohydrate), carbohydrate meals (70% carbohydrate, 15% protein, 15% fat), and fat-rich meals (50% fat, 30% carbohydrate, 20% protein).	Protein-predominant meals significantly reduced nausea and gastric slow-wave dysrhythmic activity compared to carbohydrate and fat meals (*p* < 0.05), indicating the benefit of protein-rich meals for nausea and gastric stability in early pregnancy.
[7]	Observational Study (Level III−2)	Dietary intake variations were compared in 134 pregnant women with NVP and 53 without symptoms using 3-day food diaries.	Mean protein intake in NVP group: 16.4 E% vs. 18.3 E% in non-NVP group (*p* = 0.003).	NVP was associated with significantly lower protein intake and a higher carbohydrate proportion (*p* = 0.008), indicating dietary alterations could impact maternal and fetal health.
[11]	Epidemiological Study (Level III−2)	Cross-cultural analysis of 56 studies in 21 countries, using FAO data to estimate per capita nutrient intake.	Protein intake levels not specified in grams; however, high-frequency intake of protein sources (meat, milk, and eggs consumed daily) was associated with higher NVP prevalence.	Countries with frequent protein source consumption (meat, milk, eggs consumed daily) exhibited higher NVP prevalence (*p* < 0.01), suggesting a possible adaptive biological mechanism to reduce the intake of potentially risky foods for the fetus.
[4]	Clinical Guidelines (Level IV)	Clinical guidelines for managing NVP and HG with general recommendations on protein intake without detailed g/kg measures.	Recommends protein intake distributed in small, frequent meals with lean sources, such as poultry, fish, or legumes, ideally 3–5 times daily.	Highlights the importance of maintaining balanced nutrition through lean, protein-rich meals (poultry, fish, legumes) and suggests small, frequent meals to improve tolerance in women with NVP, though specific intake quantities are not detailed.
[6]	Prospective Cohort Study (Level II)	Study on 51,675 pregnancies in Norway, using dietary questionnaires to assess NVP symptoms and dietary intake during the first trimester.	Protein intake estimated at 0.7 g/kg for the NVP group, with reduced intake in main meals; increased carbohydrates and added sugars for NVP group.	Lower protein intake refers to a reduced protein portion in main meals, with a shift toward higher carbohydrates. This adjustment in the macronutrient balance may reflect a dietary adaptation to manage NVP symptoms.
[12]	Observational Study (III−2)	Study on 303 women in the first trimester across 10 Chinese cities, using a 24 h dietary recall and semi-quantitative FFQ for a dietary intake assessment.	Women with NVP consumed approx. 15% less total protein daily, especially from meat, eggs, and dairy, compared to the non-NVP group (*p* < 0.05).	Lower protein intake indicates a daily reduction in animal-based proteins by ~15%, suggesting that reduced intake frequency and tolerance might stem from NVP symptoms, particularly at main meals.
[13]	Cross-sectional Study (Level III−2)	Analysis of dietary patterns among 3122 pregnant women using semi-quantitative FFQ and logistic regression. HG was defined based on PUQE scores and weight loss.	Protein intake from eggs, milk, seafood; sugary beverages assessed for HG risk.	Protein-rich diets (e.g., eggs, milk, seafood) reduced HG risk by 37–58%, while sugary beverages increased HG risk by 64% (*p*-trend < 0.05), highlighting a protective role of protein-rich foods against HG and the adverse effects of high-sugar diets.
[14]	Review (Level IV)	Literature review on micronutrient supplementation in pregnancy, including protein’s role in overall dietary quality.	Not specified; general discussion of protein as part of balanced nutrition.	Protein was highlighted as crucial in addressing nutritional inadequacies common during pregnancy, with supportive evidence indicating better outcomes in maternal and fetal health (*p* < 0.05).

**Table 2:** Studies reporting data regarding carbohydrates and fiber intake and NVP or HG.

Author and Year	Study Type (Evidence Level)	Methods	Dosage	Results and Conclusions
**Carbohydrates**				
[7]	Observational Study (Level III−2)	187 women, tracking dietary intake via records and interviews.	Women with NVP: 46% of caloric intake from carbohydrates vs. 41% in non-NVP.	Higher carbohydrate intake correlated with better tolerance, suggesting a high-carbohydrate diet may alleviate NVP (*p* = 0.008).
[11]	Epidemiological Study (Level III−2)	Cross-cultural analysis of 56 studies in 21 countries, using FAO data to estimate per capita nutrient intake.	High intake of simple sugars (portion or quantity not provided).	High sugar intake correlated with increased NVP prevalence (*p* < 0.001), suggesting that simple sugars might worsen symptoms.
[6]	Observational Study (Level II)	Population-based cohort of 51,675 Norwegian pregnancies, dietary intake assessed using validated food frequency questionnaires.	Women with NVP: 315.4 g/day of carbohydrates (54.3% energy) and 65.5 g/day of added sugars (11.0% energy); symptom-free group: 301.9 g/day (53.5% energy) and 59.8 g/day (10.4% energy). *p* < 0.001 for group differences.	Higher added sugar intakes (>10% energy) were observed in women with NVP compared to symptom-free women (*p* < 0.001).
**Fiber**				
[3]	Retrospective Cohort Study (Level II)	Analyzed fiber intake through pre-pregnancy and first trimester records from 188 women.	Average: 19.1 g/day pre-pregnancy, 20.6 g/day during first trimester.	Higher intake of cereal-based fiber in NVP cases (*p* = 0.043); fiber may help manage GI symptoms.

**Table 3:** Studies reporting data regarding lipid intake and NVP or HG.

Author and Year	Study Type (Evidence Level)	Methods	Dosage	Results and Conclusions
[15]	Longitudinal Observational Study (Level II)	Evaluated adherence to lipid-based nutrient supplements (LNS) in 360 Bangladeshi women. LNS intake was monitored during pregnancy and postpartum.	20 g LNS daily, containing 10 g total fats (4.59 g linoleic acid, 0.59 g alpha-linolenic acid).	Varied adherence due to GI symptoms; LNS improved maternal nutrition but severe nausea reduced tolerance.
[16]	RCT (Level I)	Analyzed morbidity symptoms from LNS intake in 1320 Ghanaian and 1391 Malawian women in two trials.	20 g/day LNS, containing essential fatty acids (4.59 g linoleic acid, 0.59 g alpha-linolenic acid).	No significant difference in symptoms (nausea, vomiting) between groups (*p* > 0.05), indicating no added benefit for NVP from LNS.

**Table 4:** Studies reporting data regarding vitamin intake and NVP or HG (excluding vitamin B6).

Author and Year	Study Type (Evidence Strength)	Methods	Dosage	Results and Conclusions
**Vitamin D**				
[17]	Prospective Study (Level II)	Studied 1500 pregnant women to assess the relationship between vitamin D intake and NVP incidence.	600–800 IU/day; adequate levels defined as serum 25(OH)D ≥ 50 nmol/L.	Women with adequate vitamin D levels reported a 15% lower incidence of NVP compared to those with deficiencies (*p* < 0.05), suggesting a protective effect.
[18]	Observational Study (Level III−2)	Analyzed vitamin D and B12 levels in a cohort of pregnant women (707 participants), assessing associations with NVP.	Varied dosages based on deficiency correction.	Adequate serum vitamin D (≥30 ng/mL) correlated with reduced NVP symptoms.
**Vitamin E**				
[19]	Systematic Review (Level I)	Reviewed vitamins E, C, B6, and B12 across multiple studies in pregnant women, focusing on NVP.	15 mg/day for antioxidant support; additional details on included study dosages.	Vitamin E showed no direct NVP effects, but author reported that its antioxidant properties may help mitigate oxidative stress linked to NVP symptoms.
**Vitamin B1 (Thiamine)**				
[20]	Retrospective Analysis (Level III−3)	Retrospective analysis of vitamin use and NVP in 1500 pregnant women; assessed impact of multivitamin use, including thiamine.	Dosage for multivitamins varied, some containing 1.4 mg/day of thiamine.	Mild reduction in NVP symptoms observed in women taking thiamine; reduction not statistically significant (*p* = 0.08).
**Vitamin B12**				
[19]	Systematic Review (Level I)	Systematic review of multiple studies on vitamins B12, C, B6, and E for pregnancy-related effects, including NVP.	~2.6 mcg/day, as recommended for neurological support.	Low B12 (below 200 pg/mL) was associated with increased NVP severity, indicating a potential benefit of adequate B12 intake for NVP management.
[18]	Observational Study (Level III−2)	Analyzed vitamin D and B12 levels in a cohort of pregnant women (707 participants), assessing associations with NVP.	Dosages adjusted individually for B12 deficiency correction.	B12 deficiency (<200 pg/mL) correlated with more severe NVP, underscoring the need for adequate B12 intake.
**Folate (Vitamin B9)**				
[21]	Randomized Controlled Trial (Level I)	Sample of 1046 women assessed the effects of folate within a multivitamin on NVP.	400 mcg/day within a multivitamin.	Women who took folic acid as part of a multivitamin reported a 12% reduction in NVP incidence (*p* = 0.02) compared to non-supplemented controls, supporting its role in nausea management.
**Riboflavin (Vitamin B2)**				
[18]	Observational Study (Level III−2)	Analyzed vitamin D and B levels in a cohort of pregnant women (707 participants), assessing associations with NVP.	Standard prenatal multivitamin dose.	Riboflavin intake showed no direct effect on NVP but may support overall metabolic health.
**Vitamin C**				
[19]	Systematic Review (Level I)	Reviewed vitamins C, B6, B12, and E across multiple studies in pregnant women, focusing on NVP and oxidative stress.	Typical daily intake of ~85 mg/day as per standard recommendations, with varying dosages across included studies.	While vitamin C did not show a specific effect on NVP, its antioxidant properties may contribute to reducing oxidative stress associated with nausea and vomiting symptoms.
[22]	Clinical Trial (Level I)	Trial on 50 pregnant women with NVP, using a chewing gum containing vitamin C to evaluate impact on nausea symptoms.	Chewing gum with 25 mg Vitamin C, administered 4 times daily.	Vitamin C gum significantly reduced nausea intensity in the treatment group compared to controls (*p* < 0.05), suggesting a potential supportive role in managing NVP.
**Multivitamin**				
[21]	Randomized Controlled Trial (Level I)	Sample of 1046 pregnant women evaluated to determine the effects of multivitamin intake on NVP.	Multivitamin containing vitamins B, C, D, and E; taken once daily.	Women taking multivitamins reported a 12% reduction in NVP incidence compared to the non-supplemented group (*p* = 0.02), supporting its protective role in NVP management.

**Table 5:** Studies reporting data regarding vitamin B6 intake and NVP or HG.

Author and Year	Study Type (Evidence Strength)	Methods	Results and Conclusions
[23]	Systematic Review and Meta-Analysis (Level I)	Included 7 RCTs with a total of 819 participants comparing ginger and vitamin B6 for NVP management.	Vitamin B6 was found to significantly improve overall NVP scores compared to ginger (SMD 0.36, 95% CI 0.06 to 0.65; *p* = 0.02). No significant differences were observed for nausea (*p* = 0.14) or vomiting scores (*p* = 0.57). Highlights vitamin B6 as more effective in overall symptom management.
[24]	Systematic Review and Meta-Analysis (Level I)	Reviewed 18 RCTs involving 2531 participants, evaluating the effects of vitamin B6 and combination therapies for NVP.	Vitamin B6 demonstrated significant efficacy in reducing nausea severity and frequency across studies, with a pooled effect size of 0.78 (95% CI: 0.26, 1.31; *p* = 0.003) for Rhode’s score and 0.75 (95% CI: 0.28, 1.22; *p* = 0.002) for PUQE score. Combination therapies, particularly with doxylamine, showed superior outcomes compared to placebo.
[25]	Randomized Controlled Trial (Level I)	Sample: 70 pregnant women <16 weeks gestation; compared ginger (1 g/day) with vitamin B6 (40 mg/day) for 4 days. Assessed using a nausea intensity scale.	Both treatments reduced nausea by 55% (*p* < 0.01), with no notable difference between groups (*p* = 0.85). Effective in mild-to-moderate nausea.
[26]	Comparative Study (Level II)	Sample: 80 pregnant women; administered ginger (500 mg twice daily) and vitamin B6 (40 mg twice daily) for one week. Nausea assessed with daily diaries.	Both interventions reduced nausea severity by 50% (*p* = 0.03), with no significant difference (*p* = 0.7). Ginger had slightly higher patient satisfaction.
[27]	Randomized Controlled Trial (Level I)	Sample: 90 pregnant women with nausea; compared vitamin B6 (30 mg twice daily) with ginger (1 g/day) over 4 days. Symptom reduction measured using PUQE score.	Vitamin B6 led to a 70% reduction in nausea, slightly outperforming ginger (60% reduction, *p* < 0.05). Both treatments safe and well-tolerated.
[28]	Matched Cohort Study (Level II)	Sample: 160 pregnant women with NVP; compared pyridoxine (10 mg/day) vs. doxylamine-pyridoxine combo. Assessed with PUQE scale over 1 week.	Doxylamine-pyridoxine combo showed 75% reduction in PUQE scores vs. 50% with pyridoxine alone (*p* < 0.01). Higher efficacy in severe cases.
[29]	Triple-Blind Randomized Trial (Level I)	Sample: 77 pregnant women in first trimester; compared ginger (500 mg twice daily), vitamin B6 (40 mg twice daily), and placebo over 4 days. Nausea assessed with Rhodes scale.	Ginger and B6 groups improved by 60% compared to placebo (*p* < 0.01), no significant difference between ginger and B6 (*p* = 0.10). Effective for mild cases.
[30]	Experimental Study (Level II)	Sample: 60 pregnant women with nausea; administered vitamin B6 supplements (10 mg daily) for 2 weeks. PUQE score used for nausea measurement.	B6 supplementation reduced nausea by 45% and vomiting by 40% (*p* < 0.05). Most effective in mild-to-moderate cases, well-tolerated by patients.
[31]	Comparative Study (Level II)	Sample: 100 pregnant women with moderate nausea; compared ginger (1 g/day) and vitamin B6 (40 mg/day) over 4 days. Assessed with a standardized nausea and vomiting scale.	Symptom improvement of 55% in both groups, with no significant difference (*p* = 0.7). B6 and ginger equally effective for mild cases.
[32]	Randomized Controlled Trial (Level I)	Sample: 291 pregnant women <16 weeks gestation; ginger (1.05 g/day) vs. vitamin B6 (75 mg/day) for 3 weeks. Assessed with the Rhodes nausea and vomiting index.	Equivalent symptom reduction in both groups (*p* = 0.8). Nausea decreased by 60% in both groups, no adverse effects reported, high tolerability.
[33]	Randomized Controlled Trial (Level I)	Sample: 342 women <17 weeks gestation; vitamin B6 (30 mg/day) vs. placebo for 5 days. Assessed nausea via visual analog scale and vomiting episodes.	Nausea severity significantly reduced in B6 group (*p* = 0.0008). Vomiting episodes also decreased but did not reach statistical significance (*p* = 0.0552). Significant efficacy for mild-to-moderate nausea.
[34]	Randomized Controlled Trial (Level I)	Sample: 256 pregnant women; doxylamine-pyridoxine (Diclectin) vs. placebo over 14 days. Assessed with PUQE scale.	Significant improvement in symptoms with Diclectin (PUQE score change −4.8 vs. −3.9, *p* = 0.006). Well-tolerated and effective for mild-to-moderate cases of NVP.
[35]	Randomized Controlled Trial (Level I)	Sample: 92 hospitalized women with HG; pyridoxine (20 mg thrice daily) vs. placebo for 2 weeks. Measured vomiting frequency and nausea scores.	No significant difference in vomiting or nausea scores between groups (*p* > 0.05). Limited efficacy observed for severe cases.
[36]	Randomized Controlled Trial (Level I)	Sample: 256 pregnant women; doxylamine-pyridoxine (10 mg each) vs. placebo for 14 days. PUQE scale used.	Modest symptom improvement with active treatment (PUQE score change −2.8 vs. −1.8, *p* = 0.01)

**Table 6:** Studies reporting data regarding mineral intake and NVP or HG.

Author and Year	Study Type (Evidence Level)	Methods	Dosage	Results and Conclusions
**Zinc**				
[37]	Randomized Controlled Trial (Level I)	Sample: 675 pregnant women in Egypt with low serum zinc levels. Groups: zinc (30 mg ZnSO4/day), zinc + multivitamin, placebo. Monitored for pregnancy complications and neonatal infections.	30 mg ZnSO4/day for zinc-only group; zinc + vitamins B1, B6, D3, C, and E for combined group.	Zinc supplementation (alone or combined) significantly reduced second- and third-stage complications, stillbirths, preterm births, and early neonatal morbidity. The effect on NVP was not specifically evaluated in this study.
[12]	Observational Study (Level III−2)	Sample: 303 Chinese women in the first trimester. Evaluated association between NVP symptoms and dietary zinc intake via 24 h recall and questionnaire.	Zinc intake levels not specified; assessed dietary intake.	Zinc intake was significantly lower in women with NVP (*p* < 0.05), suggesting a correlation between NVP symptoms and lower zinc intake, possibly linked to nutrition deficiency.
**Iron**				
[38]	Randomized Controlled Trial (Level I)	Sample: pregnant women received either iron and folic acid supplements or a multivitamin with iron. Evaluated for iron-deficiency complications.	Iron as part of multivitamin; specific dose not provided.	Iron supplementation reduced iron-deficiency complications and lowered anemia prevalence compared to control. No specific decrease in NVP symptoms.
[39]	Randomized Trial (Level I))	Sample: 180 pregnant women with anemia; compared 20, 40, and 80 mg iron doses daily for 8 weeks. Monitored for GI side effects and hemoglobin improvements.	20, 40, and 80 mg iron daily	Low-dose iron (20 mg) effectively treated anemia with fewer gastrointestinal side effects (including fewer NPV) than higher doses (*p* < 0.05). No specific effects of supplementation on NPV.
**Potassium**				
[12]	Observational Study (Level III−2)	Sample: 303 Chinese women in the first trimester. Evaluated association between NVP symptoms and potassium intake using a 24 h dietary recall and questionnaire.	Potassium intake levels not specified.	Potassium intake was significantly lower in women with NVP (*p* < 0.05), indicating a potential link between nausea and lower potassium intake, impacting the electrolyte balance.
**Calcium**				
[16]	Randomized Controlled Trial (Level 1)	Sample: 500 pregnant women in Ghana. Compared calcium intake across supplementation groups, tracking maternal and fetal outcomes.	Calcium included in daily micronutrient supplement; exact dose not specified.	Calcium supplementation led to significant improvements in maternal bone density and fetal growth (*p* < 0.05).
**Selenium**				
[12]	Observational Study (Level III−2)	Sample: 303 pregnant women in China. Examined selenium intake and NVP symptoms using 24 h dietary recall.	Selenium dosage not specified; assessed dietary intake only.	NVP group showed lower selenium intake (*p* < 0.05), suggesting that NVP symptoms may be linked to lower selenium levels.
**Phosphorus**				
[12]	Observational Study (Level III−2)	Sample: 303 pregnant women in the first trimester in China. Evaluated association of NVP symptoms with phosphorus intake.	Phosphorus intake not specified in supplement doses; dietary assessment only.	Phosphorus intake was lower in NVP cases (*p* < 0.05), indicating potential nutritional imbalance due to NVP.

**Table 7:** Studies reporting data regarding food group intake and NVP or HG.

Author and Year	Study Type (Evidence Level)	Methods	Dosage	Results and Conclusions
**Meat, Fish, and Eggs**				
[13]	Observational Study (Level III−2)	Sample of 2515 pregnant women in China; examined dietary patterns and HG risk.	High intake of fish, meat, and eggs	Diet rich in fish, meat, and eggs (3–4 servings/week of fish) was linked to a 37% reduced risk of HG (*p*-trend < 0.05), suggesting a protective effect.
[7]	Cohort Study (Level II)	Compared dietary intake between women with and without NVP.	Reduced meat intake for NVP group	NVP group consumed less meat, lower protein caloric ratio (*p* = 0.007), indicating that meat avoidance may reduce NVP symptoms.
[40]	Validation Study (III−2)	Sample: pregnant women in Japan; validated FFQ for food groups, such as meat, fish, and eggs, among NVP and non-NVP groups.	Consumption varied between groups	Higher intake of eggs and fish (2–3 times/week) among non-NVP participants (*p* < 0.05), suggesting that these foods may have a positive association with reduced NVP.
**Fruits and Vegetables**				
[41]	Validation Study (Level III−2)	Validated FVQ for fruit and vegetable intake in pregnant women, measuring FV consumption effects	Five daily servings of fruit/vegetables	Higher FV intake (at least 5 servings daily) associated with better tolerance and reduced nausea, suggesting potential benefit for NVP management.
[40]	Observational Study (Level III−2)	Examined correlations between vegetable intake and NVP among Japanese pregnant women.	Vegetable intake assessed based on FFQ	Higher intake of vegetables (3–5 servings/week) linked to lower NVP symptoms (*p* < 0.05), supporting FV intake guidelines for pregnancy.
**Grains and Fiber**				
[3]	Cohort Study (Level II)	Questionnaire assessing fiber intake and NVP symptoms among pregnant women	Cereal and vegetable fibers	Women with NVP maintained fiber intake (3–4 servings daily), suggesting fiber tolerance; steady intake could help manage symptoms.
[12]	Observational Study (Level III−2)	Dietary intake analysis comparing nutrient consumption between NVP and non-NVP groups	Dietary fiber from whole grains	Lower fiber intake in NVP group (*p* < 0.05); increasing fiber intake (3–4 servings daily) may benefit symptom management.
[40]	Validation Study (Level III−2)	Assessed relationship between cereal intake and NVP using FFQ and dietary records among Japanese pregnant women.	Cereal intake varied with NVP	Higher cereal intake (2–3 servings daily) linked to reduced NVP incidence in non-NVP group (*p* < 0.05), suggesting tolerance to grains.

**Table 8:** Studies reporting data regarding ginger extract and NVP or HG.

Author and Year	Study Type (Evidence Level)	Methods	Results and Conclusions
[23]	Systematic Review and Meta-Analysis (Level I)	Included 7 RCTs with a total of 819 participants comparing ginger and vitamin B6 for NVP management.	Ginger was effective in alleviating nausea and vomiting, showing no significant differences compared to vitamin B6 for nausea (*p* = 0.14) or vomiting (*p* = 0.57). Vitamin B6 was more effective for overall NVP scores (SMD 0.36, 95% CI 0.06 to 0.65; *p* = 0.02).
[42]	Systematic Review and Meta-Analysis (Level I)	Analyzed 13 RCTs involving 1174 participants on ginger’s effect on NVP.	Ginger significantly reduced nausea intensity across multiple studies (pooled effect size = 0.821; 95% CI, 0.585 to 1.056; *p* < 0.001). The meta-analysis revealed consistent efficacy in reducing nausea severity but found no significant effect on vomiting frequency. A high safety profile was noted, with minimal side effects reported. The study advocated for standardized dosing protocols to ensure the reproducibility of results.
[43]	Randomized Controlled Trial (Level I)	Sample: 80 breast cancer patients undergoing chemotherapy; administered ginger extract (1 g daily) vs placebo over 5 days. Evaluated using nausea intensity and frequency scales.	Ginger group showed a 60% reduction in nausea scores vs 30% in placebo (*p* < 0.05). Vomiting episodes decreased by 55% (*p* < 0.05).
[31]	Comparative Study (Level II)	Sample: 100 pregnant women with moderate nausea; compared ginger (1 g/day) and vitamin B6 (40 mg/day) over 4 days. Assessed with a standardized nausea and vomiting scale.	Symptom improvement of 55% in both groups, with no significant difference (*p* = 0.7). B6 and ginger equally effective for mild cases.
[44]	Experimental Study (Level II)	Sample: 60 pregnant women in first trimester; administered ginger biscuits (250 mg ginger/biscuit, twice daily) for one week. Assessed nausea frequency and severity.	Nausea reduced by 50% (*p* < 0.01), vomiting reduced by 45% (*p* < 0.01). Most effective for mild nausea cases, with high patient tolerance.
[45]	Randomized Controlled Trial (Level I)	Sample: 70 women with hyperemesis gravidarum; ginger capsules (250 mg, 4 times daily) compared with placebo for 4 days. Assessed using nausea and vomiting frequency scores.	Ginger group had a 70% reduction in nausea and a 60% decrease in vomiting frequency (*p* < 0.01), showing superior effectiveness over the placebo.
[46]	Comparative Study (Level II)	Sample: 120 pregnant women with nausea; compared ginger (500 mg twice daily) and dimenhydrinate (50 mg twice daily) over one week. Outcomes measured with symptom diaries.	Ginger led to a 65% reduction in nausea vs 68% with dimenhydrinate; no significant difference observed (*p* = 0.65), indicating comparable efficacy.
[32]	Randomized Controlled Trial (Level I)	Sample: 291 pregnant women <16 weeks gestation; ginger (1.05 g/day) vs vitamin B6 (75 mg/day) for 3 weeks. Assessed with the Rhodes nausea and vomiting index.	Equivalent symptom reduction in both groups (*p* = 0.8). Nausea decreased by 60% in both groups, no adverse effects reported, high tolerability.
[25]	Randomized Controlled Trial (Level I)	Sample: 70 pregnant women <16 weeks gestation; compared ginger (1 g/day) with vitamin B6 (40 mg/day) for 4 days. Assessed using a nausea intensity scale.	Both treatments reduced nausea by 55% (*p* < 0.01), with no notable difference between groups (*p* = 0.85). Effective in mild-to-moderate nausea.
[26]	Comparative Study (Level II)	Sample: 80 pregnant women; administered ginger (500 mg twice daily) and vitamin B6 (40 mg twice daily) for one week. Nausea assessed with daily diaries.	Both interventions reduced nausea severity by 50% (*p* = 0.03), with no significant difference (*p* = 0.7). Ginger had slightly higher patient satisfaction.
[27]	Randomized Controlled Trial (Level I)	Sample: 90 pregnant women with nausea; compared vitamin B6 (30 mg twice daily) with ginger (1 g/day) over 4 days. Symptom reduction measured using PUQE score.	Vitamin B6 led to a 70% reduction in nausea, slightly outperforming ginger (60% reduction, *p* < 0.05). Both treatments safe and well-tolerated.
[47]	Randomized Controlled Trial (Level I)	Sample: 120 pregnant women <16 weeks; ginger (500 mg/day) vs placebo for 4 days. Self-recorded symptoms with Rhodes Index.	Significant reduction in nausea and vomiting in ginger group compared to placebo (*p* < 0.001). Demonstrated efficacy and safety in mild-to-moderate cases.
[48]	Case-Control Study (Level II)	Sample: 65 pregnant women; ginger capsules (“Gingocap”) vs pyridoxine (30 mg/day) over 8 weeks. Symptom frequency assessed.	Gingocap reduced symptoms more effectively than pyridoxine (*p* = 0.03). High patient acceptability and no reported side effects.
[49]	Randomized Controlled Trial (Level I)	Sample: 120 pregnant women; ginger capsules (1 g/day) vs placebo for 4 days. Symptom severity measured via visual analog scale.	Ginger significantly reduced nausea severity compared to placebo (*p* < 0.001). Effective in reducing both nausea and vomiting in moderate cases.

**Table 9:** Comparison between international guidelines on nutrition during pregnancy and the reported nutritional objectives.

Nutrients	WHO Recommendations, 2016	DGA Guidelines Advisory Committee, 2020
Carbohydrates	45–60% of total energy (RNI)	45–65% of total energy (AMDR)
Simple Carbohydrates	Limit free sugars to <10% of total energy (RNI)	Reduce added sugars to <10% of total energy (AMDR)
Proteins	10–15% of total energy (RNI)	10–35% of total energy (AMDR)
Lipids	15–30% of total energy (RNI)	20–35% of total energy (AMDR)
Saturated Fatty Acids	<10% of total energy (RNI)	<10% of total energy (AMDR)
Polyunsaturated Fatty Acids	6–11% of total energy (RNI)	5–10% of total energy (AMDR)
DHA (mg/day)	200–500 mg/day (RNI)	Not specified
Fiber (g/day)	≥25 g/day (RNI)	14 g per 1000 kcal (AI)
Vitamin A (μg/day)	600–700 μg/day (RNI)	700–900 μg/day (RDA)
Vitamin D (μg/day)	5–15 μg/day (RNI)	15 μg/day (RDA)
Vitamin E (mg/day)	10 mg/day (RNI)	15 mg/day (RDA)
Vitamin K (μg/day)	55–65 μg/day (RNI)	90–120 μg/day (AI)
Vitamin C (mg/day)	45 mg/day (RNI)	75–90 mg/day (RDA)
Vitamin B1 (mg/day)	1.1 mg/day (RNI)	1.1–1.2 mg/day (RDA)
Vitamin B2 (mg/day)	1.1 mg/day (RNI)	1.1–1.3 mg/day (RDA)
Vitamin B3 (mg/day)	14 mg/day (RNI)	14–16 mg/day (RDA)
Vitamin B5 (mg/day)	5 mg/day (RNI)	5 mg/day (AI)
Vitamin B6 (mg/day)	1.3 mg/day (RNI)	1.3–1.7 mg/day (RDA)
Vitamin B7 (μg/day)	30 μg/day (RNI)	30 μg/day (AI)
Vitamin B9 (μg/day)	400 μg/day (RNI)	400 μg/day (RDA)
Vitamin B12 (μg/day)	2.4 μg/day (RNI)	2.4 μg/day (RDA)
Calcium (mg/day)	1000 mg/day (RNI)	1000–1300 mg/day (RDA)
Phosphorus (mg/day)	700 mg/day (RNI)	700 mg/day (RDA)
Magnesium (mg/day)	310–320 mg/day (RNI)	310–420 mg/day (RDA)
Iron (mg/day)	18 mg/day (RNI)	8–18 mg/day (RDA)
Zinc (mg/day)	8 mg/day (RNI)	8–11 mg/day (RDA)
Copper (mg/day)	0.9 mg/day (RNI)	0.9 mg/day (RDA)
Selenium (μg/day)	55 μg/day (RNI)	55 μg/day (RDA)

RDA: Recommended Dietary Allowance; RNI: Recommended Nutrient Intake; AMDR: Acceptable Macronutrient Distribution Range).

**Table 10:** Global recommendations for food-based components of the FIGO Nutrition Checklist Adapted from [52].

Nation	Fruit and Vegetables	Dairy Products	Wholegrains	Meat, Poultry, or Eggs
**USA**	1.5–2 servings of fruit and 2.5–3.5 servings of vegetables per day	3 servings per day	3–5 servings per day	60–100 g per day (dependent on weight), 20–25% of total calorie intake during pregnancy. Consume with every main meal.
**India**	1–2 servings of fruit and 4–5 servings of vegetables per day	3–5 servings per day	50–55% of total calorie intake. 9 portions of 30 g per day	Variable, depending on dietary patterns.
**Ireland**	5 servings per day	3 servings per day	3–5 servings per day	2 servings per day in the first and second trimesters. 3 servings per day in the third trimester.
**Canada**	Daily, consume a variety including dark green vegetables and orange vegetables	Daily. Drink fortified soy beverages if not drinking milk	Daily	Eat lean meats and alternatives daily.
**Australia**	At least 2 servings of fruit per day and at least 5 servings of vegetables per day	3.5 servings for those aged 18 years or under. 2.5 servings per day for those aged over 18 years	8 servings of carbohydrate foods per day (serving 30–40 g) for those aged 18 years or under, to 8.5 servings per day for those aged over 18 years. Choose mostly wholegrains.	3.5 servings per day.
**Kenya**	5 servings per day	Everyday	With every meal	At least 2 servings per week. Eat red meat and liver when available.
**Colombia**	2–3.5 fruit servings. 2–3 servings of vegetables	3–4 servings of dairy	3–3.5 servings of grains per day	2.5–3.5 servings of meat or poultry a day. One egg per day.

Information was gathered from various international sources as referenced. Where relevant, recommendations within the guidelines were converted into grams per day to estimate servings.

## Data Availability

The data presented in this study are available on request from the corresponding author/Supplementary Materials, further inquiries can be directed to the corresponding author.

## Supplementary Materials

The following supporting information can be downloaded at: https://www.mdpi.com/article/10.3390/foods14030373/s1.
