# Supplementary material for: A Food Pyramid and Nutritional Strategies for Managing Nausea and Vomiting During Pregnancy: A Systematic Review
Source: Foods. 2025 Jan 23;14(3):373. doi: 10.3390/foods14030373 (PMC11817518; doi:10.3390/foods14030373)
Supplement: Supplementary file 1 [file foods-14-00373-s001.zip › foods-3417394-supplementary.pdf]

APPENDIX A: WEEKLY AVERAGE BROMATOLOGICAL COMPOSITION OF A STANDARD DIET OF 2000 KCAL FOLLOWING THE NEW PYRAMIDS OF NVP AND HG IN PREGNANCY

BROMATOLOGICAL COMPOSITION

KCAL: 2022 (8460,048 Kj)
PROTEINS: 85.5 g
TOTAL FATS: 66.7 g (29.2% of total Kcal)
SATURATED FATS: 13.4 g (5.6% of total Kcal)
TOTAL CARBOHYDRATES: 52.8% of total Kcal
INTRINSIC SIMPLE CARBOHYDRATES: 9.7% of total Kcal
EXTRINSIC SIMPLE CARBOHYDRATES: 2% of total Kcal
TOTAL FIBER: 33 g
TOTAL DIETARY CALCIUM: 1.16 g
TOTAL DIETARY SODIUM: 1.8 g

Data sourced from CREA, IEO, FDA, USDA, CIQUAL, CoFID databases.

DAILY PLAN

BREAKFAST:

- **One protein portion, choose from:**
  o One jar of low-fat semi-skimmed yogurt (125 g)
  o One cup of semi-skimmed milk (150 g)
- **One carbohydrate portion, choose from:**
  o Bread (70-80 g)
  o Rusks or other dry starchy product (35 g)
  o Breakfast cereals (35 g)
- **One medium-sized fruit** (150 g)

MID-MORNING SNACK:

- **One carbohydrate portion, choose from:**
  o Bread (70-80 g)
  o Rusks or other dry starchy product (35 g)
  o Breakfast cereals (35 g)
- **One portion of nuts** (30 g)
- **One medium-sized fruit** (150 g)

LUNCH *(can be swapped with dinner for convenience)*:

- **120 g of pasta/rice/other starchy grains** (amaranth/quinoa/buckwheat/spelt/teff/sorghum), simply seasoned.
- **One protein portion** (to pair with the first course as a "condiment" or main dish), choose from:
  o Two eggs
  o 100 g of lean cured meats (e.g., prosciutto crudo or cotto, trimmed of visible fat)
  o 100 g of fresh cheese (e.g., ricotta, primo sale, light Philadelphia, crescenza, stracchino,

etc.), or 150 g of cottage cheese, or 50 g of aged cheese (e.g., Grana, Asiago, Provolone, etc.)
o 50 g of dried legumes or 150 g of canned legumes (rinsed appropriately)
o 50 g of smoked salmon or canned tuna

- **200 g of raw vegetables** (80-100 g if salad).

MID-AFTERNOON SNACK:

- **One carbohydrate portion, choose from:**
  o Bread (70-80 g)
  o Rusks or other dry starchy product (35 g)
  o Breakfast cereals (35 g)
- **One protein portion, choose from:**
  o 25 g of legumes (or hummus form)
  o One egg (60 g)
  o 50 g of low-fat cheese
  o One jar of low-fat semi-skimmed yogurt (125 g)
  o One cup of semi-skimmed milk (150 g)

DINNER (can be swapped with lunch for convenience):

• One carbohydrate portion, choose from:

- 80 g of bread
- 40 g of dry cereal-based products (e.g., rice cakes, rusks, crackers, breadsticks)
- 220 g of potatoes or other tubers (e.g., Jerusalem artichoke)

• One protein portion, choose from:

- 100 g of lean cuts of white or red meat (e.g., chicken thigh or breast, rabbit, turkey, veal fillet)
- 150 g of fresh or frozen fish (e.g., salmon, tuna steak, swordfish, sea bream, plaice, cod, hake, sole, sea bass, etc.)
- Two eggs (optionally with two additional egg whites)
- 50 g of dried legumes or 150 g of canned legumes (rinsed appropriately)

• 200 g of raw vegetables (80-100 g if salad).

THROUGHOUT THE DAY

- Every day you have **3 tablespoons or 6 teaspoons (30 ml)** of extra virgin olive oil at your disposal for cooking and dressing meals, preferably used raw.
- Grated cheese (e.g., Grana Padano, Parmigiano Reggiano, Pecorino, etc.) can be used daily on the first course at both lunch and dinner.
- If desired, it is possible to **replace one portion of oil with olives**: **10 ml of oil (1 tablespoon)** corresponds to approximately **42 g of black olives**.
- If desired, it is possible to **replace one portion of oil with avocado**: **10 ml of oil (1 tablespoon)** corresponds to approximately **30 g of avocado (a quarter of an avocado)**.
